# Clinical hematological and biochemical parameters in Swiss, BALB/c, C57BL/6 and B6D2F1 *Mus musculus*

**DOI:** 10.1002/ame2.12139

**Published:** 2020-12-18

**Authors:** Giorgio Silva‐Santana, Juliet Cunha Bax, Débora Cristina Silva Fernandes, Daniela Tendler Leibel Bacellar, Cleber Hooper, Alexandre Alves Souza Oliveira Dias, Cristina Barbosa Silva, Aline Moreira de Souza, Simone Ramos, Ricardo Alexandre Santos, Thainara Ramos Pinto, Mariana Antunes Ramão, Ana Luíza Mattos‐Guaraldi

**Affiliations:** ^1^ Health Sciences Center Institute of Microbiology Paulo de Góes Federal University of Rio de Janeiro Rio de Janeiro RJ Brazil; ^2^ Laboratory of Diphtheria and Corynebacteria of Clinical Relevance Faculty of Medical Sciences University of the State of Rio de Janeiro Rio de Janeiro RJ Brazil; ^3^ The Collaborating Centre for Reference and Research on Diphtheria/National Health Foundation/Ministry of Health Rio de Janeiro RJ Brazil; ^4^ Laboratory of Clinical Research and Molecular Diagnostic Prof. Marcílio Dias do Nascimento Department of Clinical Practice and Pathology Fluminense Federal University Niterói RJ Brazil; ^5^ Laboratory Bacterial Vaccines Department of Immunology National Institute for Quality Control in Health Oswaldo Cruz Foundation Rio de Janeiro RJ Brazil; ^6^ Institute of Science and Technology in Biomodels Oswaldo Cruz Foundation Rio de Janeiro RJ Brazil; ^7^ Institute of Biology Federal Fluminense University Niterói RJ Brazil; ^8^ Biomedical Center College of Medical Sciences University of the State of Rio de Janeiro Rio de Janeiro RJ Brazil

**Keywords:** B6D2F1, BALB/c, biochemical, C57BL/6, hematological, Swiss

## Abstract

**Background:**

Animal models are widely used in scientific research in order to obtain information from a whole organism under a specific set of experimental conditions. Various lineages of mice have been used to investigate diseases and new therapeutic strategies, and, consequently, hematological and biochemical tests in these laboratory animals are essential to validate scientific studies. Our study seeks to establish reference values for hematological and biochemical parameters of four lineages of mice.

**Methods:**

We evaluated the hematological and biochemical profiles of 20 males and 20 females from the lineages Swiss (heterogeneous), BALB/c and C57BL/6 (isogenic), and B6D2F1 (hybrid), totaling 160 mice. Analysis were standardized using the systems pocH‐100iV *Diff*™ for 19 hematological parameters and VITROS^®^ 350 for 12 biochemical parameters.

**Results:**

Results are shown as means and standard deviation, grouped by lineage and genre. Comparing the values obtained in this study with the values from previous studies, some variations were detected, which could be explained by differences in methodologies or individual variability.

**Conclusion:**

Thus our study shows that knowledge and disclosure of the values of physiological parameters of laboratory animals is necessary, and emphasises the importance of considering variations influenced by gender, lineage and genotype in the choice of the best experimental model.

## 1 INTRODUCTION

Laboratory mice are the most commonly used animal model for biological studies of human health leading to the establishment of new diagnostic and therapeutic strategies.^1^, ^2^ The demand for rat models has also increased in pharmacological, oncological and toxicological research, as well as in studies on drug efficacy,^2^ due to their easy creation, short generation time and the availability of inbred lineages (at least 20 generations of brother‐sister mating).^3^, ^4^

The extensive mapping of the mice's genome and the detailed understanding of its immunological properties^5^ have improved the standardization of experimental models, increasing the reproducibility of studies and the comparison of results by researchers worldwide, thereby minimizing the need to repeat experiments.^2^ Advances in transgenic research and genetic targeting in models that use laboratory animals have led to a deeper understanding of the mechanisms of many diseases, and revealed new possibilities for treatment in human and veterinary medicine.^1^, ^5^

Swiss heterogeneous mice (non‐consanguineous, heterogamous, outbred) present 99% heterozygosity between allele genes and therefore they have been used as a source of consanguineous animals representing natural populations and to obtain hybrid and transgenic descendants.^6^ Their aggressive behavior makes them good models in studies related to causes and/or mechanisms of aggression.^7^ This lineage is widely used for scientific purposes such as biomedical research on metabolic and autoimmune diseases, complement fixation, and mammary gland or lung tumors, as well as in pharmacology as stock in security testing for drugs.^8^ Due to their superior maternal abilities, Swiss females are used as ideal pseudogestants in the transfer of transgenic embryos and embryos of other lineages of mice.^9^

Both BALB/c and C57BL/6 lineages are isogenic (consanguineous, isogamous, inbred), obtained by crossing brothers and sisters for 20 consecutive generations, which allows the production of genetically homogeneous populations and, consequently, the need for fewer samples per experimental group.^6^ BALB/c mice have 99% homozygosity among allele genes, and are used for the production of monoclonal antibodies from plasmocytes.^6^ They have a low incidence of mammary tumors; however, they may develop other types of cancer, such as reticular neoplasias, and primary tumors in the lung and kidneys over their lifetime. This lineage has been frequently used in studies of infection with *Listeria monocytogenes* due to their great susceptibility: the mean time to death for females is three days.^10^ The C57BL/6 lineage has 6.5% of its genome originating from the *Mus spretus* species,^6^ and is widely used for conducting spontaneous and induced mutations, since it can totally express such mutations. Its main characteristics are low susceptibility to tumors and hearing loss induced by noise, and it is commonly used in studies on cardiovascular biology, development and genetics, and immunological, neurological and sensory stimuli. More specific characteristics are expressed in sublineages such as C57BL/6J, which is widely used in the production of transgenic mice. C57BL/sublineages are genetically prone to develop obesity induced by diet, diabetes type 2, atherosclerosis, high incidence of microphthalmia, reduced bone density and hereditary hydrocephalus.^11^

Unlike the other lineages, the B6D2F1 lineage is a hybrid (originally from crossing C57BL/6 [B6] females with DBA/2 [D2] males), and is heterozygous for the B6 and D2 alleles at all locations in the genome (genetic and phenotypic uniformity). The females have been used as embryo donors for microinjection in the creation of transgenic/knockout and deletion mutations, and in behavioral research, radiation and nutrient bioassays, drug and hormone research, and transplant (tumor, ovaries and skin) research using other lineages.^12^

Variations in the hematological and biochemical parameters of mice may be related to lineage, genotype, and genre, and are influenced by age, diet, environment, place of collection, and other factors.^3^, ^13^, ^14^ Therefore, knowledge of the physiological parameters and appropriate interpretation of hematological and biochemical serum rates are relevant to an evaluation of homeostasis and the alterations induced by pathological processes in different organs,^15^, ^16^ since they provide information about the animal's clinical conditions, nutritional balance, iron deficiency in hemoglobin and presence of infections, and are useful in monitoring the efficacy and the prognosis of treatments.^17^

Our study sought to establish values to serve as reference values in various hematological and biochemical of serum parameters in mice of the Swiss (heterogeneous), BALB/c and C57BL/6 (isogenic) and B6D2F1 (hybrid) lineages, to guide researchers in the best choice of an experimental model.

## 2 MATERIALS AND METHODS

### 2.1 Animal experimentation

All experimental procedures described in this study were approved by the Animal Experimentation Ethics Committee of the Institute of Biology Roberto Alcântara Gomes of the of the University of the State of Rio de Janeiro (UERJ) in accordance with Law 11.794/2008 (Arouca Law) approved in 2018 under protocol 055/2018. All procedures used are in accordance with the Brazilian Directive for the Care and Use of Animals for Scientific and Didactic Purposes of National Council for Animal Experimentation Control (CONCEA).

This study used 20 male and 20 female mice from each of the Swiss (heterogeneous), BALB/c and C57BL/6 (isogenic) and B6D2F1 (hybrid) lineages, totaling 160 individuals; with 60 days of life mice were 60 days old (age). The mice were examined and showed no lesions or inflammation of the skin and no ectoparasites at the time of collection. The animals were kept in the bioterium of the School of Medical Sciences of the UERJ, in rigorous sanitary conditions with quarter‐hourly monitoring. They were free of specific pathogens (SPF), and were housed in racks of mini isolators (5 mice/cage; cage dimensions 45 × 31 × 19 cm) lined with sterile pine wood shavings of (ASPEN – LIGNOCEL^®^), which were periodically renewed. The mice were kept in filtered air at positive pressure, with a 12 hours light/dark cycle, starting at 6:30 am, temperature ~21 to 22°C (±2°C) and humidity ~50 to 55%. All animals received sterile water (autoclaved) and appropriate rations (Nuvilab^®^ CR‐1) ad libitum, meeting the nutritional requirements for the species. The animals were not exposed to chemical or drug treatments that could change their natural physiological state.

### 2.2 Blood sampling method and sample handling

All biological material was collected by the same qualified professional. Material was collected after an 8‐hour fast, in the morning, to avoid variations in the parameters. Initially, the absolute weights of the animals were measured using a precision balance (Marte – AD 2000, maximum load capacity 210 g; sensitivity of 0.01 g). Subsequently, they were sedated by intraperitoneal injection (lower right quadrant) of 16 mg/kg of xylazine hydrochloride^®^ as a muscle relaxant and 120 mg/kg of ketamine hydrochloride^®^ for deep sedation, using BD Ultra‐Fine TM syringes (needle 25 × 5 mm). After deep anesthesia was confirmed by the absence of a foot reflex, the animals were positioned in dorsal decubitus for decontamination of the collection site with 70% ethanol. Blood was collected (intracardiac puncture – cardiocentesis) via a needle (27 × 8 mm), inserted into the abdominal wall, under the xiphoid process, according to the method of Silva‐Santana et al (2020).^18^ This procedure resulted in the death of the animals, certified by the absence of vital signs (heartbeat and respiratory movements).

Aliquots of 300 µL of blood were collected in tubes (10 × 45 mm, maximum volume 500 µL – VACUPLAST) containing ethylenediamine tetra‐acetic acid (EDTA‐K2) and carefully mixed by inversion in a homogenizer (Electra – Homolaby 22T) for a complete blood count (CBC). For biochemical analysis of serum, aliquots of blood were deposited in tubes (10 × 45 mm, maximum volume 500 µL – VACUPLAST) containing separator gel and coagulation activators, to the maximum volume indicated by the manufacturer, waiting 30 minutes to blood clot retraction. The aliquots were then centrifuged for 5 minutes at 2500 rpm (Eppendorf^®^ Minispin^®^ model SPIN 1.000, Hamburg, Germany) to separate the serum. The biological samples were then sent to the Institute of Science and Technology in Biomodels, Fundação Oswaldo Cruz, Rio de Janeiro, Brazil.

### 2.3 Hematological parameters

CBC was performed in an automated veterinary hematology counter pocH‐100*iV Diff*™ (Sysmex^®^ ‐ Roche) to establish the following parameters: red blood cell (RBC) count, hemoglobin concentration (HGB), hematocrit (HCT), mean corpuscular volume (MCV), mean corpuscular hemoglobin (MCH), mean corpuscular hemoglobin concentration (MCHC), red cell distribution width‐coefficient of variation (RDW‐CV), red blood cell dimension width‐standard deviation (RDW‐SD), number of white blood cells (WBC), percentage ratio of small white blood cells (W‐SCR), percentage ratio of middle white blood cells (W‐MCR), percentage ratio of large white blood cells (W‐LCR), number of small white blood cells, lymphocytes (W‐SCC), number of middle white blood cells, monocytes (W‐MCC), number of large white blood cells, gametocytes (W‐LCC), number of platelets (PLT), mean platelet volume (MPV), platelet dimensions width (PDW), percentage of giant platelets (P‐LCR).

Hematoscopy was performed on blood smears colored with instant coloration Leucognost^®^, by immersion microscopy (Leica DM500, Wetzlar, Germany; 1.000×) to confirm the results obtained in the CBC and for differential analysis of the leukocytes (counts of lymphocytes (LYM), monocytes (MON), eosinophils (EOS), neutrophils – segment or immature (bands) (NEU – SEG/BAN) and basophiles (BAS)), evaluation of blood cell morphology and photographic documentation. The equipment was calibrated and the precision of results determined before each analysis using control blood ABX Minotrol 16, and an external quality evaluation program was implemented.

### 2.4 Clinical biochemistry parameters

The blood serum was processed in an automated spectrophotometer VITROS^®^ 350 Chemistry System (Ortho‐Clinical Diagnostics, Johnson‐Johnson Co., Rochester, NY) for analysis of the following parameters: total protein (TP), triglycerides (TG), cholesterol (CHL), alkaline phosphatase (AP), uric acid (UA), alanine transaminase (ALT), aspartate transaminase (AST), albumin (ALB), globulin (GLB), glucose (GLC), creatinine (CR) and urea (UR). The biochemical kits and calibration controls used were acquired from Labtest Diagnosis (Lagoa Santa, Minas Gerais, Brazil), and were used according to protocols established by manufacturer.

### 2.5 Statistical analysis

The variables of interest monitored were absolute body weights and the values obtained by hemogram and serum biochemistry in all four lineages of mice (Swiss, BALB/c, C57BL/6 and B6D2F1) of both genders (male and female). The data obtained for analysis were reported as mean values and standard deviation (SD), with 95% confidence intervals (CI); *P* > .05 was considered statistically significant. The statistical analysis was conducted using software GraphPad Prism version 6.01.

## 3 RESULTS

Male mice presented higher values of total body weight compared to females of the same age. The animals tested showed no evidence of hemoparasites in the cytological evaluation of blood smears by microscopy. The analysis of erythrocytes revealed a greater number of RBC in females; the other parameters varied according to lineage and gender (Table 1). Higher PLT counts were found in C57BL/6 and B6D2F1 males, while the opposite was observed in Swiss. Male and female mean PLT counts were similar in BALB/c mice (Table 2).

**Table 1:** Reference intervals for erythrocyte indices in healthy mice of mixed breeds

Erythrogram								
Parameters (Unit)	SWISS (mean ± SD)		BALB/c (mean ± SD)		C57BL/6 (mean ± SD)		B6D2F1 (mean ± SD)	
	Male	Female	Male	Female	Male	Female	Male	Female
BW (g)	35.55 ± 2.66	29.35 ± 1.19	27.03 ± 1.39	20.80 ± 0.67	27.02 ± 1.22	20.01 ± 2.20	37.43 ± 1.36	32.75 ± 1.51
RBC (×10^6^/µL)	7.42 ± 0.85	7.74 ± 0.69	9.00 ± 0.44	9.08 ± 0.62	8.45 ± 1.15	8.86 ± 1.01	8.70 ± 1.17	7.99 ± 0.69
HCT (%)	39.81 ± 1.64	40.74 ± 1.71	43.52 ± 2.89	42.57 ± 3.68	41.91 ± 3.41	37.14 ± 3.46	46.19 ± 5.67	42.73 ± 2.20
HGB (g/dL)	12.92 ± 0.34	13.45 ± 0.71	13.64 ± 0.74	13.82 ± 1.07	13.13 ± 0.97	12.01 ± 1.02	13.89 ± 0.99	13.03 ± 0.15
MCV (fL)	57.81 ± 7.79	54.56 ± 5.17	49.23 ± 1.98	47.73 ± 4.26	50.74 ± 3.18	42.69 ± 8.51	53.09 ± 0.69	54.09 ± 1.94
MCH (pg)	18.58 ± 2.03	17.52 ± 2.21	15.56 ± 1.01	14.48 ± 1.04	16.23 ± 1.38	14.10 ± 2.56	16.19 ± 1.06	16.59 ± 1.28
MCHC (g/dL)	31.78 ± 0.97	32.57 ± 1.02	31.77 ± 1.50	33.00 ± 2.60	31.03 ± 2.04	28.94 ± 1.24	30.43 ± 1.61	30.59 ± 1.19
RDW‐CV (%)	15.64 ± 1.47	16.55 ± 1.72	16.34 ± 1.22	14.47 ± 0.88	15.33 ± 0.71	14.33 ± 1.60	20.80 ± 4.10	21.99 ± 5.29
RDW‐SD (fL)	29.23 ± 0.75	31.16 ± 2.80	28.90 ± 2.11	27.30 ± 2.97	31.55 ± 1.40	29.09 ± 3.25	36.04 ± 4.75	39.20 ± 7.61

Note

CBCs were performed in an automated veterinary hematology counter pocH‐100*iV Diff*™ (Sysmex^®^ ‐ Roche). Data show means ± standard deviation (SD), calculated with GraphPad Prism version 6.01.

Abbreviations: BW, body weight; HCT, hematocrit; HGB, hemoglobin concentration; MCH, mean corpuscular hemoglobin; MCHC, mean corpuscular hemoglobin concentration; MCV, mean corpuscular volume; RBC, red blood cell; RDW‐CV, red cell distribution width ‐ coefficient of variation; RDW‐SD, red blood cells dimension width ‐ standard deviation.

**Table 2:** Reference intervals for platelets indices in healthy mice of mixed breeds

Plaquetogram								
Parameters (Unit)	SWISS (mean ± SD)		BALB/c (mean ± SD)		C57BL/6 (mean ± SD)		B6D2F1 (mean ± SD)	
	Male	Female	Male	Female	Male	Female	Male	Female
PLT (×10^3^/µL)	0.418 ± 0.080	0.626 ± 0.087	0.560 ± 0.358	0.560 ± 0.119	0.171 ± 0.056	0.120 ± 0.034	1.249 ± 0.013	1.056 ± 0.080
MPV (fL)	6.16 ± 0.14	9.79 ± 2.43	7.38 ± 0.79	5.70 ± 1.64	8.05 ± 1.74	6.50 ± 0.89	4.83 ± 1.32	6.79 ± 0.19
PDW (fL)	7.61 ± 0.58	10.96 ± 0.71	7.58 ± 0.57	6.23 ± 1.72	9.79 ± 2.11	7.67 ± 1.08	6.23 ± 1.32	8.44 ± 0.52
P‐LCR (%)	—	—	—	—	—	—	—	—

Note

CBCs were performed in an automated veterinary hematology counter pocH‐100*iV Diff*™ (Sysmex^®^ ‐ Roche). Data show means ± standard deviation (SD), calculated with GraphPad Prism version 6.01.

Abbreviations: —, absence of data; MPV, mean platelet volume; PDW, platelet dimensions width; P‐LCR, percentage of giant platelets; PLT, number of platelets.

The automated leukocyte count showed that BALB/c females have a greater number of immune cells. However, the differential leukometry data showed that the male Swiss mice had higher rates of W‐SCC (LYN) compared to other lineage of mice and the females had higher rates of W‐MCC (MON) and W‐LCC (NEU). In the cytological evaluation of blood smears by microscopy, the highest numbers of LYN and MON were found in females and males, respectively, in the B6D2F1 lineage, the highest numbers of EOS were found in C57BL/6 females, and NEU were highest in BALB/c males (Table 3).

**Table 3:** Reference intervals for leukocyte indices in healthy mice of mixed breeds

Leukogram								
Parameters (Unit)	SWISS (mean ± SD)		BALB/c (mean ± SD)		C57BL/6 (mean ± SD)		B6D2F1 (mean ± SD)	
	Male	Female	Male	Female	Male	Female	Male	Female
Global leucometry								
WBC (×10^3^/µL)	4.98 ± 1.26	5.16 ± 1.02	5.36 ± 0.96	6.23 ± 2.57	5.80 ± 0.34	4.24 ± 0.74	3.84 ± 0.23	5.99 ± 0.48
W‐SCR (%)	83.43 ± 1.28	92.46 ± 6.22	75.66 ± 6.37	66.58 ± 26.19	84.95 ± 7.04	80.01 ± 5.79	81.59 ± 6.04	85.89 ± 4.21
W‐MCR (%)	12.46 ± 1.12	13.15 ± 1.88	17.33 ± 1.99	13.29 ± 6.69	13.25 ± 1.16	13.94 ± 1.56	14.30 ± 3.81	10.26 ± 2.61
W‐LCR (%)	4.45 ± 1.04	4.82 ± 1.41	7.37 ± 3.24	5.55 ± 1.55	5.08 ± 0.44	6.74 ± 1.05	4.14 ± 2.27	3.73 ± 1.61
W‐SCC (×10^2^/µL)	6.57 ± 0.97	5.59 ± 0.75	3.88 ± 1.02	3.59 ± 0.76	5.25 ± 0.50	4.16 ± 0.39	3.19 ± 0.40	5.13 ± 0.15
W‐MCC (×10^2^/µL)	1.01 ± 0.18	1.24 ± 0.37	1.01 ± 0.35	0.85 ± 0.19	0.75 ± 0.15	0.80 ± 0.10	0.59 ± 0.11	0.69 ± 0.19
W‐LCC (×10^2^/µL)	0.39 ± 0.11	0.50 ± 0.19	0.46 ± 0.21	0.43 ± 0.11	0.29 ± 0.08	0.40 ± 0.09	—	—
Differential leucometry								
LYM (×10^2^/µL)	7.65 ± 0.63	6.86 ± 0.97	6.60 ± 0.64	7.27 ± 0.85	7.28 ± 0.55	5.02 ± 0.33	7.45 ± 0.63	7.99 ± 0.19
MON (×10^2^/µL)	2.72 ± 0.73	3.10 ± 0.87	2.52 ± 1.29	2.98 ± 1.79	2.30 ± 0.57	1.99 ± 0.50	3.29 ± 0.31	3.10 ± 0.29
EOS (×10^2^/µL)	1.15 ± 0.55	1.39 ± 0.34	1.49 ± 0.94	1.88 ± 0.98	2.18 ± 0.70	4.19 ± 0.83	1.99 ± 0.31	1.30 ± 0.29
NEU/SEG (×10^2^/µL)	1.39 ± 0.34	1.69 ± 0.27	3.25 ± 0.57	2.16 ± 0.64	2.73 ± 0.41	2.12 ± 0.45	1.62 ± 0.40	1.59 ± 0.11

Note

CBCs were performed in an automated veterinary hematology counter pocH‐100*iV Diff*™ (Sysmex^®^ ‐ Roche). Data show means ± standard deviation (SD), calculated with GraphPad Prism version 6.01.

Abbreviations: —, absence of data; BAS, basophiles cells; EOS, eosinophils; LYM, lymphocytes; MON, monocytes; NEU/BAN, absence of immature neutrophils (bands); NEU/SEG, neutrophils/Segmented; WBC, number of white blood cells; W‐LCC, NEU, number of large white blood cells, gametocyte; W‐LCR, percent ratio of large white blood cell; W‐MCC, MON, number of middle white blood cells, monocyte; W‐MCR, percent ratio of middle white blood cell; W‐SCC, LYN, number of small white blood cells, lymphocyte; W‐SCR, percent ratio of small white blood cell.

**FIGURE 1:**
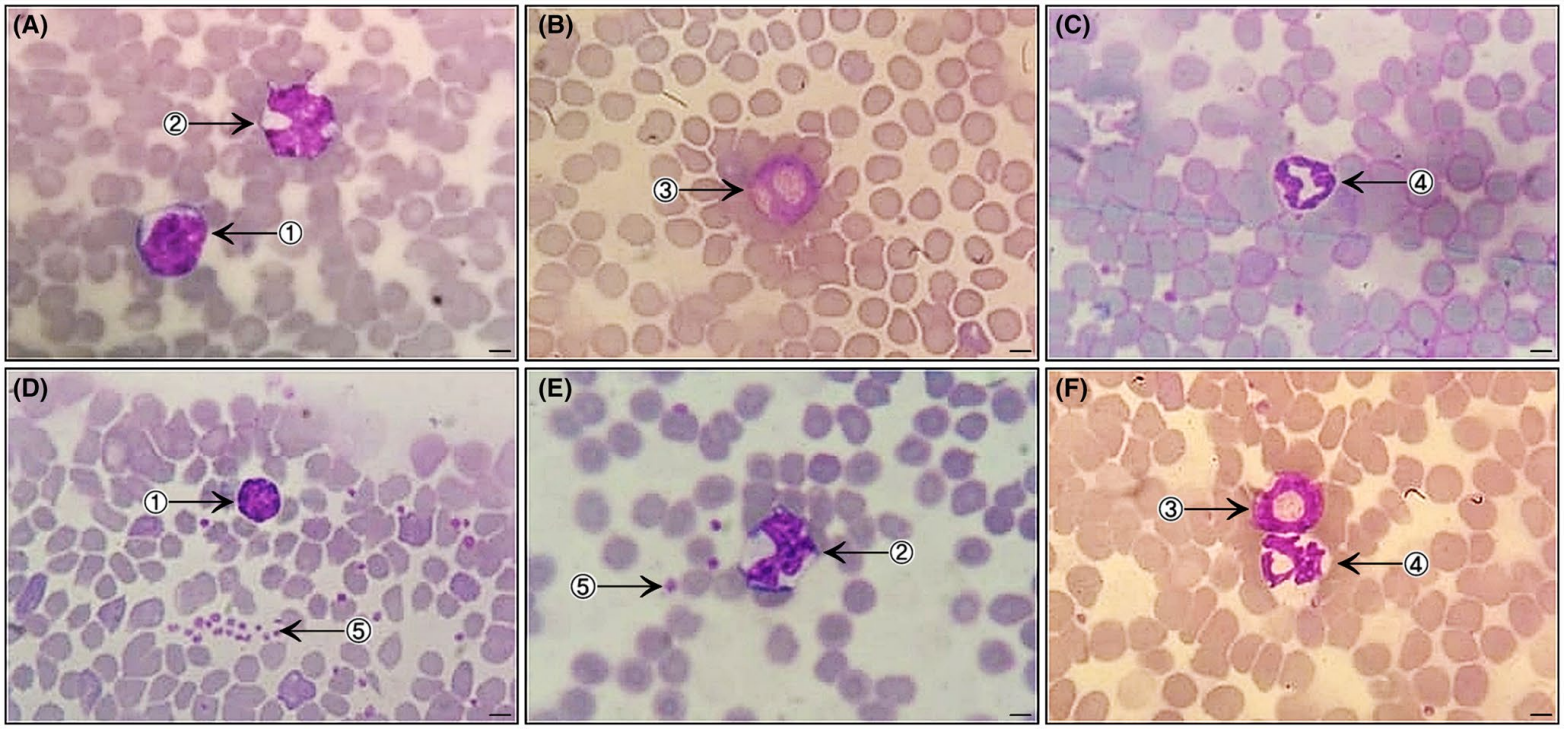
Photomicrographs of bloods smears (Diff‐Quick staining; 1000×; bar = 500 µm) from healthy mice: Swiss female (A and B) and male (C); BALB/c male (D and E) and female (F). The smears show red blood cells present in large quantities with uniform distribution, and evidence of leukocytes: lymphocyte (1), monocyte (2), eosinophil (3), neutrophil (4) and platelets (5)

**FIGURE 2:**
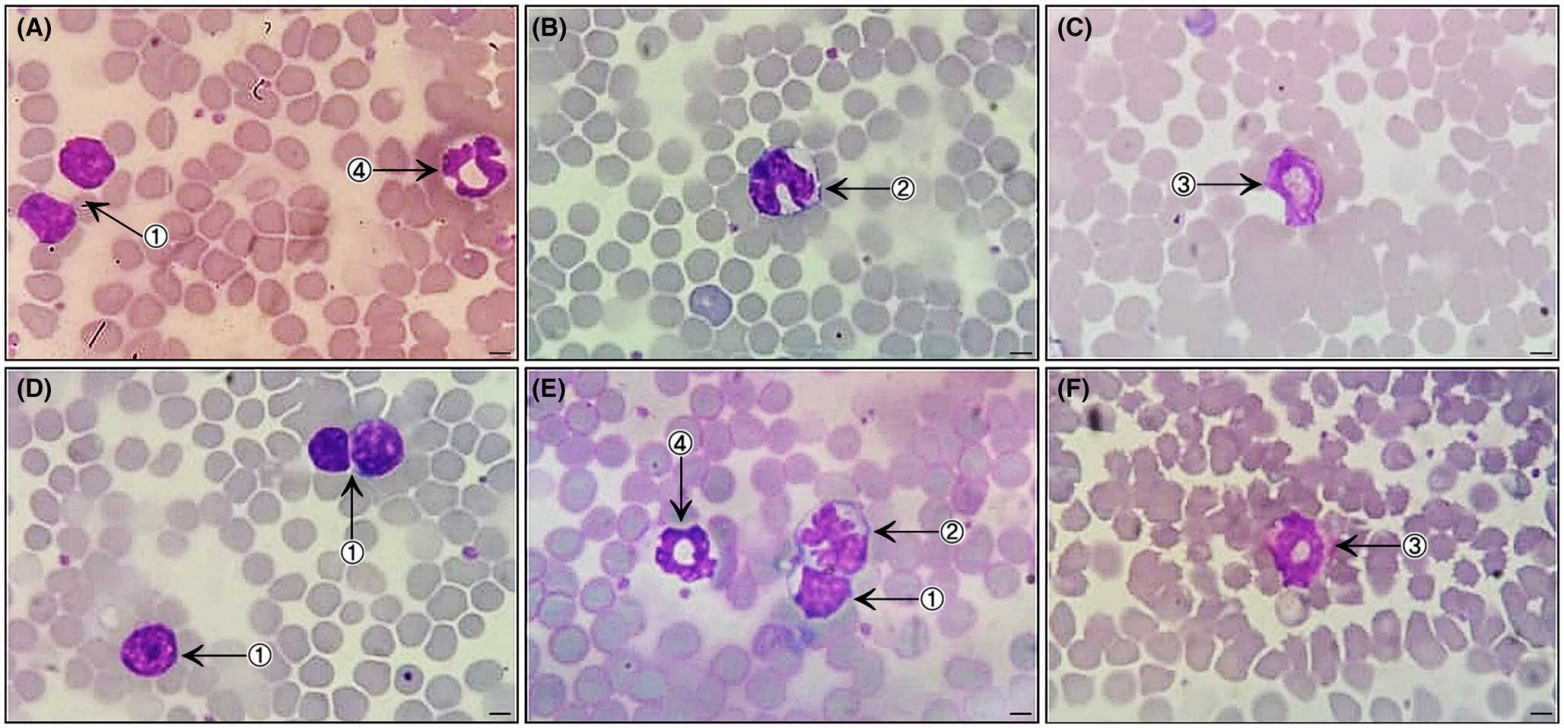
Photomicrographs of bloods smears (Diff‐Quick staining; 1000×; bar = 500 µm) from healthy mice: C57BL/6 male (A and B) and female (C); B6D2F1 female (D) and male (E and F). The smears show red blood cells present in large quantities with uniform distribution, and evidence of leukocytes: lymphocyte (1), monocyte (2), eosinophil (3), neutrophil (4)

Blood cell morphology of did not differ among the lineages studied. Erythrocytes presented as anucleate biconcave discs with central pallor. A few polychromatophilic erythrocytes and some cells containing Howell‐Jolly corpuscles, which are common aspects in some species, were observed. Lymphocytes and neutrophils were the most commonly observed leukocytes. Regarding morphology, lymphocytes showed a rounded nucleus with condensed chromatin and reduced cytoplasm, monocytes were kidney‐shaped and ovoid, with clearer chromatin and almost imperceptible granulation in the cytoplasm, eosinophils presented with a bilobulated nucleus and cytoplasm filled with orange granules, and neutrophils presented with a polylobulated nucleus and discrete fine basophilic granules dispersed in abundant cytoplasm. Immature forms of neutrophils were not observed. Anucleate and slightly flattened platelets were observed, as well as few platelet aggregates (Figures 1 and 2).

The blood serum was limpid and semi‐transparent in aspect. The analyses of biochemical parameters demonstrated higher ALB and UR levels in Swiss males; higher CHL levels in BALB/c males, and higher AP and ALT in females; higher TP and UA levels in C57BL/6males, and higher AST and GLB in females; and finally, higher TG levels in B6D2F1 males and higher GLC levels in females (Table 4).

**Table 4:** Reference intervals for serum biochemical indices in healthy mice of mixed breeds

Serum biochemistry								
Parameters (Unit)	SWISS (mean ± SD)		BALB/c (mean ± SD)		C57BL/6 (mean ± SD)		B6D2F1 (mean ± SD)	
	Male	Female	Male	Female	Male	Female	Male	Female
UR (mg/dL)	60.54 ± 6.46	40.69 ± 4.63	40.48 ± 3.91	36.24 ± 3.42	57.66 ± 3.16	60.15 ± 6.95	58.51 ± 10.18	39.29 ± 1.48
ALT (UI/L)	46.15 ± 5.62	59.03 ± 9.58	99.44 ± 39.61	239.50 ± 141.20	76.37 ± 20.26	110.00 ± 16.83	65.72 ± 6.95	73.79 ± 9.46
AST (UI/L)	121.60 ± 35.93	163.60 ± 48.13	135.20 ± 26.53	156.70 ± 57.20	136.70 ± 34.86	293.40 ± 62.65	90.09 ± 8.12	95.23 ± 16.25
AP (UI/L)	169.20 ± 32.56	147.30 ± 23.58	209.10 ± 36.21	362.90 ± 226.60	97.29 ± 9.58	106.00 ± 16.83	249.80 ± 6.28	119.00 ± 8.27
TP (g/dL)	5.31 ± 0.31	5.14 ± 0.47	5.21 ± 0.46	5.23 ± 1.68	8.03 ± 0.34	7.85 ± 0.62	4.42 ± 0.17	5.60 ± 0.53
ALB (g/dL)	2.77 ± 0.17	1.84 ± 0.37	2.40 ± 0.47	1.74 ± 0.50	2.35 ± 0.12	2.10 ± 0.47	2.29 ± 0.12	2.33 ± 0.06
GLB (g/dL)	2.60 ± 0.23	3.34 ± 0.28	2.77 ± 0.23	3.50 ± 1.89	5.80 ± 0.31	5.94 ± 0.40	2.19 ± 0.08	3.30 ± 0.56
UA (mg/dL)	2.32 ± 0.28	1.32 ± 0.20	2.19 ± 0.24	2.55 ± 0.24	2.67 ± 0.61	1.09 ± 0.10	1.89 ± 0.21	1.43 ± 0.16
CHL (mg/dL)	90.20 ± 6.13	91.81 ± 8.51	129.10 ± 11.82	77.72 ± 30.33	77.73 ± 3.22	44.92 ± 6.18	102.00 ± 6.64	66.03 ± 5.32
TG (mg/dL)	140.40 ± 15.36	94.02 ± 3.29	76.89 ± 22.42	68.11 ± 34.57	31.56 ± 2.12	31.56 ± 2.42	214.40 ± 12.49	187.80 ± 11.52
GLC (mg/dL)	147.60 ± 31.76	214.40 ± 47.29	160.40 ± 26.21	141.90 ± 30.47	233.20 ± 4.65	208.80 ± 6.95	239.80 ± 22.13	242.40 ± 9.62

Note

The Creatinine (CR) showed values below the detection limits (<0.2 mg/dL). Blood sera were processed at VITROS^®^ 350 (Ortho Clinical Diagnostics ‐ Johnson & Johnson) automated spectrophotometer. The Mean and Standard Deviation (SD), calculated through GraphPad Prism version 6.01.

Abbreviations: ALB, albumin; ALT, alanine transaminase; AP, alkaline phosphatase; AST, aspartate transaminase; CHL, cholesterol; GLB, globulin; GLC, glucose; TG, triglycerides; TP, total protein; UA, uric acid; UR, urea.

## 4 DISCUSSION

The evaluation of hematological and biochemical phenotypes is relevant to determining the physiological profile of different lineages and/or populations of mice, as well as allowing the creation of reference values that will form the basis of the interpretation of variations caused by diseases.^14^

Mice are the most commonly used animals in experiments. However, there are controversies over the use of species and subspecies created in laboratories due to the presence of special intersections, in which the animals have some genes or even chromosomes of other species.^19^ Therefore, knowledge of the hematological and biochemical phenotypes of the lineages produced in the laboratory and used in research is important.^5^

Hematological parameters are used as biomarkers in the diagnosis of organ or tissue injuries and as an aid in animal reproduction, and in diagnosing infections, parasitosis and other pathologies. In clinical practice, the parameters commonly used to evaluate the erythrogram are the RBC, HCT, HGB, MCV, MCH and MCHC counts, and less frequently RDW counts (CV and SD).^20^ HGB is of extreme importance in electron transport, and reduction and transfer of oxygen for hydroxylation reactions; it can be measured as the total volume in the erythrocytes, and has a value of ~10 to 17 g/dL in mice.^21^ The dataset obtained for RBC, MCV, MCH and MCHC enables an analysis of the correlation between the size of erythrocytes and the HGB concentration in its interior, which is important for characterizing different degrees of anemia.^22^ MCV is used to evaluate the degree of anisocytosis (size of erythrocytes) and in the classification of anemia among normocytic, microcytic or macrocytic erythrocytes.^22^, ^23^ MCH can be estimated from the relationship between the mean concentration of hemoglobin inside of the cell and the number of erythrocytes, which may vary between ~13 and 17 pg in mice.^22^ MCHC measures hemoglobin concentration in erythrocytes, and values range between ~30 and 38 g/dL in mice.^21^ RDW evaluates the range of variation in size of erythrocytes in the same sample of blood, through a histogram of the distribution of erythrocytes according to volume. Different disorders may either increase or decrease this parameter.^24^, ^25^

RBC and MCHC were highest in BALB/c females among the groups studied. Similar RBC data were presented by Santos et al (2016) (8.90 ± 0.90 × 10^6^/µL) for collections by puncture in the axial plexus, and similar MCHC data were reported by Spinelli et al (2012) (32.24 ± 0.58 g/dL) for collections by puncture in the submandibular vein. B6D2F1 males presented higher indices of HCT and HGB, and B6D2F1 females of RDW (CV and SD). No comparable studies in hematology have used mice of this lineage. Swiss males presented the highest MCV and MCH values, which were similar to those presented by Diniz et al (2006) for mice produced by TACONIC, an animal model company (MCV, 58.5 ± 0.65 fL and MCH, 18.58 ± 2.03 pg). No other studies have used RDW as a parameter for profiling lineages of mice, which is strange, since the RDW index changes early, even before reductions in HGB and MCV, demonstrating its great importance in identification of anisopoikilocytosis.^24^, ^25^ The morphology of heritocytes was similar among the lineages studied, as reported in previous studies.^26^ The morphological changes in the erythrocytes were not observed in our study, that is, the cells were normal and had no changes.

Currently, many automated hematological counters provide different parameters for platelet analysis. Despite still being little understood, they are used in medical and laboratory practice, particularly because of the difficulty in standardization. In analogy to the erythrogram and leukogram, the parameters that analyze the platelets composed of PLT, PDW, MPV and P‐LCR came to be called plateletogram.^27^

The platelets are anucleate fragmented cells derived from megakaryocytes. They have hemostatic (buffer) function, and maintain endothelium integrity by releasing pro‐angiogenic cytokines.^28^ These cells can be activated spontaneously or in response to stimuli, depending on lineage activation stage.^21^ In mice, platelet count is ~900 to 1600 × 10^3^/mm^3^.^29^ Analysis of the association between MPV and PDW is used to determine the distribution, width and size of platelets. Disturbances may result in variations in pre‐established parameters.^27^, ^29^ The P‐LCR measurement is clinically important in identifying rare hereditary diseases such as macrothrombocytopenia (abnormally large platelets).^27^ B6D2F1 males showed a higher PLT level among the groups studied. No previous studies have used this lineage. Swiss females presented the highest MPV and PDW values. Despite their greater sensitivity as a clinical marker of platelet function and activity compared with PLT, we could not find any comparable studies containing analyses of these parameters.^29^ High levels of these indices indicate the existence of large platelets, which are more metabolically and enzymatically active. On the other hand, large platelets are more aggregative, facilitating thrombus formation, and are related to vascular diseases, including of the coronary artery.^27^, ^29^ We could not measure P‐LCR in the lineages studied, possibly due to the small sample volume (<12 fL), indicating that animals in the selected lineages had no macroplatelets. Evaluation of platelet morphology by blood smears is also part of the complete blood count, and this showed a normal aspect in our study, as well as a normal count.

Understanding immunological characteristics is essential to evaluating inherited or acquired disorders.^30^ Leukocytes participate in immune and inflammatory processes, being responsible for mediating the innate and adaptive immune response. In mice, the total WBC count is ~2 to 10 × 10^3^/mm^3^ and, together with the parameters W‐SCR, W‐MCR and W‐LCR, it can be quantified automatically or manually. The differential leukocyte count provided by automated hematological counters is based solely in the size of these cells and must be always confirmed by microscopic analysis of a blood smear.^21^, ^31^

BALB/c females showed higher WBC rates than other lineages. Similar values were obtained by Spinelli et al (2014) (5.88 ± 1.40 × 10^3^/µL), who collected the blood by puncture of the retro‐orbital vein plexus; males of the same lineage showed high W‐MCR and W‐LCR values. Swiss females showed higher W‐SCR rates. We could not find any comparable studies using automated assessments of small, medium and large white blood cells.

LYNs are mostly mediators of adaptive immunity,^17^ and comprise ~ 70% to 80% of leukocyte counts in mice, which can increase to above 80% in young animals.^21^, ^31^ MON comprise ~0% to 2% of the total leukocyte count in mice^21^, ^31^ and, along with free macrophages and those fixed in the tissues, are responsible for initiating the immune response, and phagocytozing and destroying microorganisms such as bacteria.^32^ Our automated and microscopic evaluation of lymphocytes and monocytes showed higher W‐SCC and MON values in males of the Swiss and B6D2F1 lineages and higher W‐MCC and LYN values in females. EOS represent ~0% to 7% of the total count of leukocytes in mice,^21^, ^31^ and, together with BAS, are mediators of the pathogenesis of many inflammatory processes against helminthic infections and allergic diseases.^33^ C57BL/6 females showed higher rates of EOS. Lower values were obtained by Spinelli et al (2012) (2.60 ± 3.10 × 10^2^/µL). NEU are circular phagocytes that regulate immune responses, and constitute the first line of defense against invasion by microorganisms, trauma to tissues or any inflammatory signals.^34^ They represent 0% to 30% of the total count of leukocytes in mice.^21^, ^31^ Swiss females showed higher W‐LCC values. BALB/c males, on the other hand, presented higher NEU/SEG values; however, higher values were obtained by Spinelli et al (2014) (11.00 ± 4.93 × 10^2^/µL).

Blood counts of leukocytes undergo constant changes (leukocytosis), which may indicate infectious and/or inflammatory conditions, myeloproliferative and lymphoproliferative disorders (lymphosarcoma and leukemia) and tissue necrosis due to severe infection. When there is a medullar deficit, leukocyte production is reduced (leukopenia) and the organism is vulnerable to pathogens. The most common causes of medullar deficit are viral diseases, massive bacterial infections, anaphylaxis, endogenous toxemia (uremia) and neoplasia of medulla bone.^35^

The determination of biochemical parameters provides important information on clinical status, nutritional balance, and the metabolic functioning of the organs and tissue, as well as evidence of occult diseases, enabling the monitoring of treatment and prognosis.^36^, ^37^

Clinical evaluation of renal function is based on measurement of UR, together with CR.^38^, ^39^ Generated in the liver, UR is the main nitrogen metabolite derived from protein degradation^39^; 90% is excreted by the kidneys, ~40% to 70% returns to plasma.^38^ Mice at eight weeks of age have, on average, 0.28 mg/dL CR,^40^ derived from creatine catabolism. CR is present in large amounts in the skeletal and cardiac muscles, liver and kidneys, and is excreted by the kidneys, especially by glomerular filtration.^41^ Swiss males presented the highest UR indices. Branco et al (2011) reported similar values (53.00 ± 1.90 mg/dL) using brachial plexus bleeding. However, we could not measure CR in the animals tested, possibly because its concentration was below the minimum (<0.2 mg/dL) required for detection by the automated system. Hypothetically, the highest CR rates should be expressed in males of the B6D2F1 and Swiss lineages, since they have higher body weights than the other lineages and variations in serum concentration are intimately related to body weight and mass and muscle metabolism.^14^

The assessment of hepatic function in response to anatomical or biochemical alterations^42^ is commonly subjected to dosage testing of ALT, AST, AP and TP.^43^ The origin of ALT is predominantly plasmatic; it is found abundantly in the liver, in moderate amounts in the kidneys and in small quantities in the heart and skeletal musculature.^43^, ^44^ AST is an intracellular enzyme present in the cytoplasm and mitochondria^43^ in various organs and tissues, including liver, kidneys, heart, brain, skeletal muscle and erythrocytes.^45^ In seven‐week‐old mice, ALT and AST concentrations are, on average, of 41 U/L and 152 U/L, respectively.^46^ AP is present in the liver (epithelial cells of bile duct), bones (osteoblasts), intestines, kidneys and placenta, and is composed of a group of membrane‐associated isoenzymes located in various tissues; however, only AP present in bone and hepatobiliary tissue are important for diagnosis.^14^, ^36^, ^44^ The evaluation of TP is an important determinant of metabolism homeostasis.^47^ Proteins are found in all components of cells, being fundamental to their structures and functions^48^ and are commonly evaluated in conjunction with ALB and GLB.^47^ In six‐week‐old mice, the concentrations of AP and TP are, on average, 86 U/L and 5.22 g/dL, respectively.^49^ In our evaluation of the parameters related to the liver, BALB/c females presented higher ALT and AP indices than other lineages. Barbosa et al (2017) obtained lower values (29.72 ± 4.40 UI/L and 2.32 ± 0.85 U/L, respectively) in samples collected by cardiocentesis. C57BL/6 females presented higher indices of AST and C57BL/6 males of TP, did not obtain data for AST in C57BL/6 female mice in the literature. However, Almeida et al (2008) obtained lower results for TP in males of the same lineage (243.08 ± 51.13 g/dL) in samples collected by puncture of the retro‐orbital vein plexus.

Changes to the ALT, AST, AP and TP can be symptomatic of some diseases. The rapid elevation of ALT indicates hepatic lesion.^43^, ^44^ When associated with an increase in AST concentration, it indicates a profound damage from hepatocytes.^43^ In small animals, increased AP is observed in inflammatory and degenerative disorders of the skeletal musculature, hepatocellular dysfunction, and heart diseases (eg ischemia, congestion, necrosis, neoplasia, trauma).^45^, ^50^

ALB and GLB are important in maintaining osmotic pressure, to avoid blood extravasation, and are commonly evaluated in conjunction with TP as determinants of metabolism homeostasis.^47^ The plasma reduction of both may have similar causes to a decrease in TP.^45^, ^51^ ALB is synthesized in the liver and comprises 50% of total protein in the serum. It is responsible for 80% of colloidal osmotic pressure and acid‐base equilibrium (in metabolic acidosis).^52^ GLB indices are obtained from the difference between TP and ALB; they are divided in alpha, beta and gamma globulins, and are present in inflammatory and/or infectious processes.^51^ Swiss males showed higher ALB indices than other lineages. Diniz et al (2006) reported similar values (2.78 ± 0.12 g/dL). C57BL/6 females presented higher GLB indices. We could not find comparable studies in this lineage.

Despite being rarely used in hepatic evaluation, UA is formed mainly in the liver as a final product of purine catabolism.^53^ C57BL/6 males presented greater UA indices than other lineages. Almeida et al (2008) reported lower UA indices (1.54 ± 0.68 mg/dL). High levels of UA (hyperuricemia) can result from genetically determined metabolic imbalances, such as enzyme activity and deficiency in renal excretion, leading to deposition of urate crystals in the articulation (gout), and cardiovascular diseases such as arterial hypertension.^53^

CHL and TG lipid indices can vary due to their origins, which can be exogenous (consequence of the diet) or endogenous (produced by the liver). CHL produced by the liver acts as a precursor to steroid hormones.^51^ TG can be formed in the cells of the intestinal mucosa, from monoglycerides and long‐chain fatty acids absorbed in alimentary ingestion (exogenous); they can also be formed in the liver, and transported by blood as low‐density lipoproteins (LDLP) (endogenous).^54^ BALB/c males presented higher CHL indices than other lineages. Santos et al (2016) described similar values (135.00 ± 6.00 mg/dL). B6D2F1 males presented higher TG indices. We could not find comparable studies mentioning parameters for this index using this lineage.

GLC present in the serum is obtained mainly through the diet, and to a lesser extent from hepatic glycogen. Its main function is to generate energy (adenosine triphosphate – ATP) for biological functions.^55^, ^56^ B6D2F1 females showed higher GLC indices than other lineages. Similar studies reporting GLC values using this lineage were not found.

Our results and the comparisons with previous studies provide evidence of the existence of variations among lineages and between genres of mice. These variations must be considered during the selection of animals for experimentation, in the evaluation and observation of the results obtained, and in the analysis of the modifications resulting from induced or spontaneous pathological processes.

## 5 CONCLUSION

The determination of values for hematological and biochemical parameters in Swiss, BALB/c, C57BL/6 and B6D2F1 mice is important in the choice of experimental model and study design because of the significant differences in these parameters between lineages, genders and routes of blood collection. Automated analyses should always be confirmed by microscopy evaluation and compared with the data from the literature for optimal interpretation. Each bioterium should establish its own reference values, since a wide range of variations in physiological parameters can be due to the conditions to which the animals are subjected, and this can affect the results of research. It is important to consider the many variables that directly interfere with metabolism and, consequently, hematological and biochemical values: species, age, genetic variation and the environmental conditions to which the animals are subjected, namely temperature, relative humidity, ventilation, lighting, noise, manipulation, feeding, water, microbiota, presence of pathogens and contact with other animals.

## CONFLICT OF INTERESTS

The authors declare that there are no conflicts of interests.
